# Energy-Efficient Cooperative Spectrum Sensing Using Machine Learning Algorithm

**DOI:** 10.3390/s22218230

**Published:** 2022-10-27

**Authors:** Qingying Wu, Benjamin K. Ng, Chan-Tong Lam

**Affiliations:** Faculty of Applied Sciences, Macao Polytechnic University, Macao SAR, China

**Keywords:** cognitive radio, cooperative spectrum sensing, multi-dimensional optimization, neural network

## Abstract

Cognitive Radio (CR) is a practical technique for overcoming spectrum inefficiencies by sensing and utilizing spectrum holes over a wide spectrum. In particular, cooperative spectrum sensing (CSS) determines the state of primary users (PUs) by cooperating with multiple secondary users (SUs) distributed around a Cognitive Radio Network (CRN), further overcoming various noise and fading issues in the radio environment. But it’s still challenging to balance energy efficiency and good sensing performances in the existing CSS system, especially when the CRN consists of battery-limited sensors. This article investigates the application of machine learning technologies for cooperative spectrum sensing, especially through solving a multi-dimensional optimization that cannot be readily addressed by traditional approaches. Specifically, we develop a neural network, which involves parameters that are integral to the CSS performance, including a device sleeping rate for each sensor and thresholds used in the energy detection method, and a customized loss function based on the energy consumption of the CSS system and multiple penalty terms reflecting the system requirements. Using this formulation, energy consumption is to be minimized with the guarantee of reaching a certain probability of false alarm and detection in the CSS system. With the proposed method, comparison studies under different hard fusion rules (‘OR’ and ‘AND’) demonstrate its effectiveness in improving the CSS system performances, as well as its robustness in the face of changing global requirements. This paper also suggests the combination of the traditional and the proposed scheme to circumvent the respective inherent pitfalls of neural networks and the traditional semi-analytic methods.

## 1. Introduction

Many spectrum resources are assigned and licensed as a result of the quick development of wireless applications [1]. At any given moment and place, there exists a large portion of the valuable spectrum being unusable, which suggests that spectrum scarcity is brought on by spectrum management practices rather than a physical restriction on the range of frequencies that can be used. Solving the problem of efficient use of the spectrum is critical to the development of wireless communications. Unlicensed users may use licensed spectrum bands that are vacant as long as the primary user does not suffer undue inconvenience, as stated in the Federal Communications Commission’s (FCC) Spectrum Allocation Policy [2], which can alleviate the inefficiency problem in spectrum utilization.

With the emergence of cooperative sensing, Cognitive Radio Sensor Network (CRSN) [3], which combines Wireless Sensor Networks (WSN) and Cognitive Radio (CR) technologies [4] may dynamically detect the spectrum usage in various radio environments and improve the detection performance, as well as address the issues of WSN coexistence and spectrum underutilization. The process of detecting the idle spectrum is called spectrum sensing [5]. More specifically, performing spectrum sensing can help detect the spectra that are not occupied by licensed users (PUs, Primary Users), and allow unlicensed users (SUs, Secondary Users) to use idle licensed spectra (Spectrum Holes).

Sensors are always anticipated to have a longer lifespan, which is also essential in WSN. Less energy usage has been demonstrated to increase device longevity [3,6], thereby how to save energy consumption is a pressing issue worthy of investigation in the CSS system.

Severe noise and fading may arise in non-cooperative spectrum sensing, in which a single SU ascertains the state of the PU. This represents that the SU’s assessment of the PU status might be inaccurate, and lead to the busy main network being utilized [7]. In CSS, this issue can be solved by cooperatively distributing SUs over a Cognitive Radio Network (CRN), as the decision of the PU state can be jointly generated by the perception results of each SU and the decision criteria.

During the spectrum sensing process, the CRs cooperate to detect the activities of the PU. Local detection results are reported to the fusion center (FC), which later integrates all local decisions and gives a final decision. Considering that energy consumption is mainly concentrated on two tasks, transmission and sensing, a combined sleeping and censoring scheme [8] has been proposed to achieve a high level of energy savings. The sleeping rate can control the CRs’ working state and the censoring policy filter out local decisions that need not be transmitted to the fusion center.

Previous works have shown that under the constraints of global detection probability and global false alarm probability [9], the energy efficiency of the system can be formulated as a joint optimization problem, i.e., finding the optimal values of decision threshold and sleeping rates consuming the least energy, which does reduce the energy consumption of the whole CSS system [10,11]. Due to the complexity of the joint optimization problem, certain assumptions, such as a flat-fading environment with all sensors having equal SNR, are needed in order to reduce the size of numerical searches in finding the optimal solution.

ML-based techniques provide an attractive option for tackling such complex problems with reduced effort and time [12]. There have been ML-based previous works where solving the energy efficiency problem of distributed cooperative sensing becomes a search for the smallest subset of sensors with efficient topology. This is done by using Q-learning to instruct learning sensor selection strategies for energy efficiency by embedding data from graph structures into a neural network [13].

In this paper, we proposed a novel way to utilize ML-based technique to determine system parameters such as sleeping rate and the censoring rate to improve energy efficiency, while relaxing the assumption that all sensors have equal SNR in the CRSN, thus allowing a more realistic radio environment. The main contributions of this paper are summarized as follows.

- With the premise of a high global detection probability and a low global false alarm probability, a set of parameters for the CSS algorithm which lowers the energy consumption of the distributed sensor networks can be determined using ML.
- A neural network is constructed to select the appropriate sleeping rates and thresholds of energy detection for sensor systems with different SNRs. A custom loss function is introduced that quantifies the performance of spectrum sensing.
- Under the ‘OR’ and the ‘AND’ fusion rules, performances with the proposed method are compared with that of the traditional method.

The remainder of the paper is organized as follows. Some related works are discussed in Section 2. The CSS system model with the combined censoring and sleeping scheme is introduced in Section 3. We shall analyze the underlying optimization problem and propose the solution with machine learning algorithm in Section 4, followed by numerical results in Section 5. We shall draw our conclusions in Section 6.

## 2. Related Works

The main focus of this paper is cooperative spectrum sensing (CSS) and a brief overview of the latest development in CSS is provided in this section. In general, CSS is classified into centralized, cluster-based, and decentralized sensing. Decentralized CSS is not necessarily reliable with no fusion center. Less information is transferred between the fusion center and CR users as a result of centralized CSS, but data processing time takes longer. Cluster heads in cluster-based CSS require additional energy to process data [14].

As multiple CR users cooperate to complete spectrum sensing in CSS, the process can be divided into four stages: (1) Spectrum sensing by single nodes [15,16], (2) Each node reports its local result, (3) Fusion center (FC) fuses all nodes’ results and gives out a decision, and (4) Fusion decision broadcasted.

Compared to the other two most popular detection techniques, i.e., matched filter and cyclostationary feature detection, energy detection was adopted widely in the local spectrum sensing process because of its lowest computational and implementation complexity [17,18]. The energy detection method involves calculating the signal’s energy at a given time, comparing it to one (or more) predetermined thresholds, and determining the detection result. This distinguishes two categories of local decision-making techniques: (1) a single threshold and (2) multiple thresholds. More commonly, SUs just report PU present (received energy larger than the threshold) or absent (received energy less than the preset threshold) using the single threshold, so that the unknown noise in real-world contexts always has an impact on accuracy. The multi-threshold approach includes a ’no decision’ condition that lessens the impact of unidentified noise on spectrum sensing. After acquiring sensing information, the quality of each node’s sensing can be measured by $P_{d}$ and $P_{f}$.

The simplest procedure for sharing the sensing information is to deliver the local binary decision, which is also known as the hard decision with just one bit for representation. It is easily adopted due to its conciseness and easy-to-decode property. In contrast, in the soft-decision reporting scheme, information with an assumed level of precision is encoded and shared, which makes it precise but computationally expensive with significant data overhead and processing complexity. Each sensing node can contribute to the global quality decisions $Q_{d}$ and $Q_{f}$ for the whole CSS system [19] by sharing information. The soft-decision reporting can also be effectively used to improve the overall detection quality. As an illustration, Ref. [20] introduced an ideal soft-decision scheme based on the Neyman-Pearson criterion.

A mechanism for deciding licensed spectrum utilization in a cooperative spectrum sensing network based on local decisions made by cognitive radio users is called the fusion rule, sometimes referred to as the decision rule. The fusion rule is also divided into two different categories: hard fusion rules (OR-rule, AND-rule, and Majority-rule) and soft fusion rules (square-law selection, maximal ratio combining, square-law combining, and selection combining). Many analogies between hard and soft decisions have been made. According to [9], cooperative spectrum sensing works better than non-cooperative spectrum sensing at low signal-to-noise ratios. Additionally, Ref. [9] discussed the CSS performance analysis using the ‘AND’ and ‘OR’ rules, respectively. CSS based on soft fusion performs noticeably better than that based on hard fusion. However, the overhead of energy use is also significant [21] because of the intricate processing and massive data transmission. Ref. [22] contrasted the performance of cooperative spectrum sensing between hard fusion and soft fusion. It demonstrates that energy detection-based hard fusion rules are not necessarily superior to soft fusion techniques, while generating the soft-based decision rule’s outcome would typically need extensive computation and the expenditure of enormous amounts of energy [23,24].

In view of the delicate time allocation between spectrum sensing and spectrum access, energy consumption can be controlled by adjusting the sensing time and the spectrum-access time. For the system performance, the sensing time affects sensing accuracy, while the spectrum-access time impacts SU efficiency. Both have an effect on energy efficiency. For instance, joint optimization of the transmission stage and the spectrum sensing phase (spectrum access) is suggested by the authors of [25]. They demonstrate the existence of an optimal ratio between the sensing period and the access period that ensures the best channel efficiency. Similarly, a scheme that combines sleeping and censoring has been put up in order to achieve a significant amount of energy savings [9], which has been verified in Zigbee [11]. The transmission and perception tasks are the two main duties taken into account in the scheme. The censoring scheme is also taken into consideration in [17,26,27] in the distributed detection sensor networks. With the ‘OR’ rule [28], a censoring mechanism helps lower the communication overhead of the cognitive radio network. In order to construct the sensing parameters for a censored truncated-sequential-sensing scheme, the maximum average energy consumption per sensor is reduced in [24] according to a certain detection performance restriction. Ref. [29] considers censoring for collaborative spectrum sensing in a case where cognitive radios use cyclostationary detection as their sensing method. The combined censoring and sleeping scenario outlined in [30] is identical to the one in this work. To identify the ideal censoring and sleeping rates, the total network energy consumption is minimized while adhering to a particular detection performance restriction.

Some studies concentrated on the individual energy consumption of sensors and attempted to reduce the maximum average energy consumption per sensor rather than the total energy consumption of the network. As an illustration, Ref. [30] evaluated the issue using the ‘OR’ rule, and [10] evaluated the issue using both the ‘OR’ rule and the ‘AND’ rule. There is a joint sensing and decision node selection strategy taken into account in [31], whose network energy consumption is minimized subject to a detection performance limitation described in [30]. The research on network throughput optimization for energy-constrained cognitive radios aims to identify the best hard fusion techniques for distributed spectrum sensing without energy-efficient algorithms [32].

Based on the above discussion, we decide, in order to reduce the energy consumption as well as the computational burden of the CSS system, to adopt the hard-decision fusion rule with the sleeping and censoring scheme discussed in [9]. In [10,11], the authors implemented the combined sleep and censor scheme under ‘OR’ and ‘AND’ fusion rules in CSS, assuming that all sensors enjoy the same average SNR. As such, they are able to formulate the energy efficiency as a joint optimization problem and derive the optimal decision thresholds and sleeping rates which reduce the average energy consumption of each sensor. However, in a realistic radio environment in which the amount of fading and noises differs among sensors, the number of parameters to be optimized increases extraordinarily as each sensor shall employ different setting such as the sleeping rate. This in turn creates a multidimensional optimization problem not easily resolved by analytical means or exhaustive searches. In this paper, we aim to utilize machine learning to resolve this problem.

In the field of machine learning, solving a multi-dimensional optimization problem can be viewed as finding an optimal mapping from the inputs to the optimal solutions. The optimal mapping in machine learning can be discovered in a number of methods. The objective function for an unsupervised learning problem or an optimization problem can be set equal to the loss function [33,34]. Both unconstrained continuous optimization problems [35] and unconstrained discrete combinatorial optimization problems [36,37] can be solved via reinforcement learning (RL). Deep learning (DL) can always be used to solve optimization tasks while enforcing simple restrictions by applying generic equality and inequality constraints to optimization problems [38].

Previous works utilizing ML algorithms have yielded some good results in CSS. To balance the energy consumption among the cluster heads (CHs) in cognitive radio sensor networks, an unequal clustering technique with a DL-based algorithm has been implemented in cluster-based CSS [39]. Deep Cooperative Sensing (DCS) [40], the combination of deep neural network (DNN) and CSS, can even achieve higher sensing accuracy than traditional methods without considering the decision type and explicit mathematical formulation. The spectrum access scheme in CRSN can be characterized as the well-known i.i.d. multi-armed bandit model in Reinforcement Learning (RL), so as to maximize system throughput with the unknown environment knowledge for cognitive users [41]. By applying RL, a dynamic scanning preference list of channels can be established at each SU by using the Q-learning approach in CSS [42], which further leads to a significant performance improvement in terms of scanning overhead, access delay, and detection efficiency. While these ML-based works are promising in different aspects, none of them has considered an energy-efficiency problem of a lightweight sensor network with a single fusion center and energy-detection-based sensors adopting simple censoring and sleeping scheme, to the best knowledge of the authors. As a result, our work is unique in the sense that we solve the energy-efficiency problem of CSS by formulating a constrained minimization problem that can be resolved by the ML algorithm.

In this paper, we use the neural network to solve the aforementioned energy minimization problem in CSS in ways similar to [33,34]. By exploiting the neural network and the learning capability, we are able to solve the multi-dimensional optimization problem for a realistic and complicated CSS system environment where not all sensors share the same SNR and parameters such as sleeping rate. Although the CRSN network topology and routing are assumed to be static in this paper, our methodology can potentially be used for other CRSN topologies or energy-saving schemes, which will be a subject of future work.

## 3. Model and Problem Formulation

### 3.1. CRSN Model with a Combined Sleeping and Censoring Scheme

**Figure 1:**
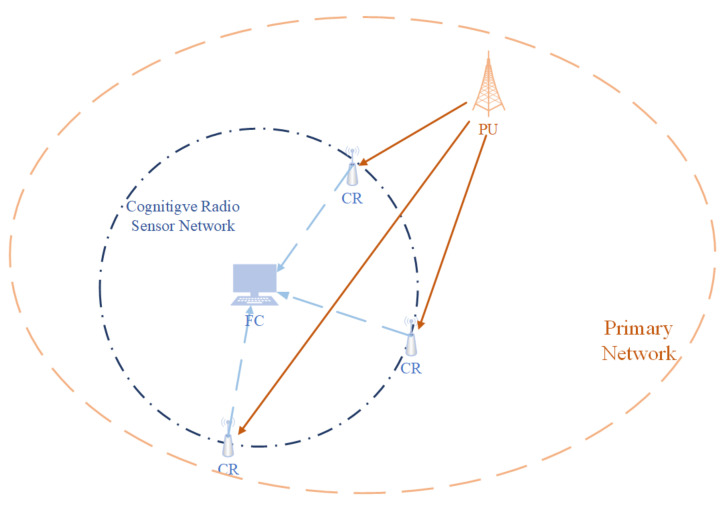
This is the architecture of the cognitive radio sensor network. It consists of a Fusion center (FC) and *M* Cognitive Radios/sensors. Each sensor will collect samples from *N* time slots and send local decisions to the FC. FC is responsible for making the final decision.

For a network containing *M* cognitive radios or sensors, each of which collects *N* signal samples $\left(i=1,\ldots ,N;j=1,\ldots ,M\right)$, the architecture of the cooperative spectrum sensing is shown in Figure 1. At the *i*-th sample of the *j*-th sensor, given the channel gain $h_{j}$, the primary user signal $s_{i}$, and the Additive White Gaussian noise (AWGN) $w_{ij}$, the received signal $r_{ij}$ depends on the presence $\left(H_{1}\right)$ or the absence $\left(H_{0}:r_{ij}=w_{ij}\right)$ of the PU, which are given by

(1) $$\left\{\begin{array}{c} H_{1}:r_{ij}=h_{j}s_{i}+w_{ij},\\ H_{0}:r_{ij}=w_{ij}. \end{array}\right.$$

As in [11], the channel gain takes the form a multiplicative coefficient due to the assumption of a quasi-static flat-fading channel. This is also the case in a frequency-selective fading channel when OFDM is employed and each subcarrier experiences a multiplicative channel gain. We remark that our energy-detection-based method will work for other channel models but for simplicity, the above channel model simplifies the computation of system performance and the simulation studies. As such, the energy detector can accumulate energy acquired from *N* samples at each CR. The energy accumulated at the *j*-th sensor is denoted as $E_{j}$ which is given by

(2) $$E_{j}=\sum_{i=1}^{N}\frac{r_{ij}^{2}}{w_{ij}^{2}}$$

The local spectrum sensing compares the test statistics of the locally received signal with thresholds. Local decision techniques are divided into two categories: (1) Single threshold, and (2) Multiple thresholds. The single-threshold approach is more common, where SUs can only report PU present (Send1, when received energy is greater than a threshold) or absent (Send0, when received energy is less than a threshold). To suppress the effect of unknown noise in real-world environments, we consider double thresholds $\lambda_{1}$ (lower threshold) and $\lambda_{2}$ (upper threshold) in constructing a local decision policy, which forms no local decision when SU is uncertain about PU’s state. The status of PU can be inferred by comparing $E{}_{j}$ with thresholds:

(3) $$\left\{\begin{array}{ccc} send\;1,\;declaring\;H_{1} & \; & if\;E_{j}\geq \lambda_{2}\\ no\;decision, & \; & if\;\lambda_{1}<E_{j}<\lambda_{2}\\ send\;0,\;declaring\;H_{0} & \; & if\;E_{j}\leq \lambda_{1} \end{array}\right.$$

The local probability of false alarm $P_{f,j}$ and the local probability of detection $P_{d,j}$ are used to evaluate the accuracy of the *j*-th CR’s local decision in spectrum sensing. Without prior knowledge of the PU signal and i.i.d. additive white Gaussian noise, they are chi-square functions that can be shown as follows [11]:

(4) $$P_{f,j}=Pr\left(E{}_{j}\geq \lambda_{2}|H_{0}\right)=\frac{\Gamma \left(N,\frac{\lambda_{2}}{2}\right)}{\Gamma \left(N\right)}$$

(5) $$P_{d,j}=Pr\left(E{}_{j}\geq \lambda_{2}|H_{1}\right)=\frac{\Gamma \left(N,\frac{\lambda_{2}}{2\left(1+\gamma_{j}\right)}\right)}{\Gamma \left(N\right)}$$

where $\gamma_{j}$ is the averaged SNR at the *j*-th CR, and Γ(a,x) is the incomplete gamma function given by $\Gamma \left(a,x\right)=\int_{0}^{\infty }t^{a-1}e^{-t}dt$, with Γ(a,x)=Γ(a). The subscript *j* is used to denote the *j*-th CR. After introducing a combined sleeping and censoring rate, assuming a priori probability of $H_{0}$ and $H_{1}$ which can be represented as $\pi_{0}=Pr\left(H_{0}\right)$ and $\pi_{1}=Pr\left(H_{1}\right)$ respectively, the censoring, i.e., no decision was made by the *j*-th CR, can be expressed as $\rho_{j}=Pr\left(\lambda_{1}<E_{j}<\lambda_{2}\right)=\pi_{0}\sigma_{0,j}+\pi_{1}\sigma_{1,j}$, where $\sigma_{0,j}$ and $\sigma_{1,j}$ are censoring rates under $H_{0}$ and $H_{1}$, respectively. They can be expressed as follows [11]:

(6) $$\sigma_{0,j}=\frac{\Gamma \left(N,\frac{\lambda_{1}}{2}\right)}{\Gamma \left(N\right)}-\frac{\Gamma \left(N,\frac{\lambda_{2}}{2}\right)}{\Gamma \left(N\right)}$$

(7) $$\sigma_{1,j}=\frac{\Gamma \left(N,\frac{\lambda_{1}}{2\left(1+\gamma_{j}\right)}\right)}{\Gamma \left(N\right)}-\frac{\Gamma \left(N,\frac{\lambda_{2}}{2\left(1+\gamma_{j}\right)}\right)}{\Gamma \left(N\right)}$$

Next, let $\mu_{j}$ denote the sleeping rate of the *j*-th CR which refers to the fraction of time the device is in the sleeping mode and not performing any detection nor communication with FC. Then, the global decision, denoted as $D_{FC}$, made by the FC is based on the specific fusion rule. The FC under the ‘AND’ rule only recognizes $D_{FC}=1$ when all local decisions are ’send 1’. While under the ‘OR’ rule, the FC will consider that $D_{FC}=1$, once one local decision is ’send 1’. The global probability of detection $Q_{d}$ and the global probability of false alarm $Q_{f}$ under both two rules are derived using the same approach in [11], as shown in Table 1, where

(8) $$Q_{d}^{Rule}=Pr\left(D_{FC}=1|H_{1}\right)=1-Pr\left(D_{FC}=0|H_{1}\right)$$

(9) $$Q_{f}^{Rule}=Pr\left(D_{FC}=1|H_{0}\right)=1-Pr\left(D_{FC}=0|H_{0}\right)$$

and the superscript Rule is given by OR or AND.

For a system of M cognitive radios, let $C_{s}$ and $C_{t}$ denote the sensing energy in *N* samples and the transmission energy per bit decision per unit distance, respectively. Under the combined sleeping and censoring scheme, no sensing energy will be consumed when sensors are sleeping, and there is no transmission energy consumed when censoring, i.e, no decision results. As such, the energy consumption of the *j*-th CR is composed of the sensing energy and the transmission energy, which can be represented as $C_{j}=\left(1-\mu_{j}\right)\left[NC_{s}+C_{t}\left(1-\rho_{j}\right)\right]$ [11]. The total energy consumed by the system, denoted as *C*, is the sum of the energies consumed by each CR, which is simply given by $C=\sum_{j=1}^{M}C_{j}$.

### 3.2. Problem Formulation

The problem we need to tackle is, on the basis of satisfying the constraints of two global probabilities in the existing CSS model mentioned above, to jointly determine the optimal values of the sleeping rates, $\mu_{j},j=1,2\ldots M$, and thresholds, $\lambda_{1}$, and $\lambda_{2}$, so that the minimum amount of total energy consumption *C* is yielded. The joint optimization problem, which is an extension of the one in [11] based solely on a single sleeping rate, is formulated as

(10) $$\begin{array}{c} \begin{array}{c} min\\ \mu_{1},\ldots ,\mu_{M},\lambda_{1},\lambda_{2} \end{array}C\\ s.t.Q_{f}\leq \alpha ,Q_{d}\geq \beta \end{array}$$

where $C=\sum_{j=1}^{M}C_{j}$, α and β are the upper limit of the global false alarm probability and the lower limit of the global detection probability respectively.

We remark that the above formulation is general so as to apply in different fading environments and location distribution of sensors in the CRSN. As seen from the expression of $C_{j}$, its parameters such as transmission energy can be arbitrary and they can be adjusted according to the sensors and the channel conditions between them and the FC. Once the values of these parameters are fixed, the joint optimization can then be solved to find the optimal sleeping rates and thresholds via the proposed ML algorithm described next.

## 4. Proposed Machine Learning Algorithm and Dataset

### 4.1. Proposed Machine Learning Algorithm

As explained before, the energy-efficient problem can be translated into the task of finding the optimal values of parameters to achieve a high probability of global detection and a low probability of global false alarm while minimal energy will be consumed. In the traditional semi-analytic method [11], the energy efficiency problem of a CSS system with multiple CRs can be readily solved by assuming that all sensors have identical signal-to-noise ratios, followed by minimizing the energy consumption of every CR. However, due to the spatial distribution of the sensors, the channel gain and local noise level from the PU signal transmitter to various sensors vary. For a CRSN with multiple CRs, we seek to find the optimal sleeping rate for each CR and the two thresholds in energy detection.

#### 4.1.1. Neural Network Structure Design

Due to the multilayer feedforward architecture, a neural network with a single hidden layer and a finite number of neurons had been regarded as capable of universal approximation [43,44]. Despite this, it is relatively inefficient compared to the solution with multiple hidden layers [45]. Yet, only with complex datasets involving time series or computer vision, more than two hidden layers can be helpful to learn more complex representations. In general, the neural network with 1 input layer, 2 hidden layers, and 1 output layer is competent for the task mentioned. Furthermore, since each sensor plays the same role in the CRSN with similar channel fading statistics, the neural network design will follow a more generic structure. It is expected that by exploiting specific CRSN topology and different fading statistics, further improvement can arguably be made in the neural network design and it is left to future investigation.

**Figure 2:**
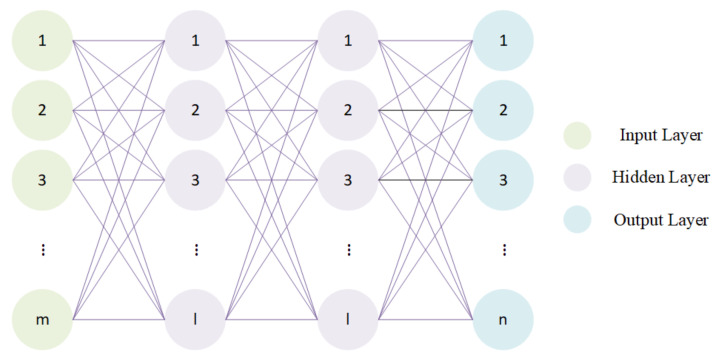
Structure of the designed neural network. The number of nodes in the input layer, two hidden layers, and output layer is *M*, *l*, *l*, and *n* respectively.

Taking *M* SNRs as inputs, the designed neural network is expected to output two thresholds and *M* sleeping rates. So, the number of nodes in the input layer and the output layer is *M* and M+2 respectively. The number of neurons in the hidden layer is determined using the empirical formula:

(11) $$l=\sqrt{m+n}+a$$

where *n* is the number of input neurons, *m* is the number of output neurons, and *a* is a constant with a value range of $\left[0,10\right]$. We remark that the empirical formula (11) is merely a rule-of-thumb based on heuristics and similar rules exist in the literature [46] when it comes to solving new and unseen problems or problems from a new domain. We consider the optimization problem being one of its kind in CSS and as a first attempt, we therefore adopt such a rule. The structure of the designed neural network is shown in Figure 2.

The following work is based on this neural network with a structure of 1 input layer, 2 hidden layers, and 1 output layer.

#### 4.1.2. NN-Based Constrainted Optimization for Spectrum Sensing

We propose that such a multi-dimensional constrained optimization problem can be transformed into a regression problem with multiple constraints.

In the designed neural network, we use a generic method originating from unsupervised learning to deal with the constrained optimization problem: the neural network is trained to minimize the customized loss function which comprises the objective function of the optimization problem and the penalty of constraint violation. Detail of using neural network to solve similar constrained optimization problems can be found in [34]. Specifically, for the combined censoring and sleeping CSS scheme, the goal is to minimize the total energy consumption *C* assuming that $Q_{f}\leq \alpha$ and $Q_{d}\geq \beta$. Therefore, the loss function can be set equal to the objective function with penalty terms added to the objective function as follows:

(12) $$L=\theta_{1}MSE+\theta_{2}C+\theta_{3}\Pi_{1}\left(Q_{d}\right)+\theta_{4}\Pi_{2}\left(Q_{f}\right)$$

where

(13) $$C=\sum_{j=1}^{M}\left(1-\mu_{j}\right)\left[NC_{s}+C_{t}\left(1-\rho_{j}\right)\right]$$

(14) $$\Pi_{1}\left(Q_{d}\right)=\left\{\begin{array}{c} 0,Q_{d}\geq \beta \\ \varepsilon ,Q_{d}<\beta \end{array}\right.$$

(15) $$\Pi_{2}\left(Q_{f}\right)=\left\{\begin{array}{c} 0,Q_{f}\leq \alpha \\ \varepsilon ,Q_{f}>\alpha \end{array}\right.$$

and ε is an ideal large number to represent the penalty of violating the constraint (ideally infinity), which is set to be 100 in the paper. Considering the same importance among the four terms in (8), their weights are set to be $\theta_{1}=\theta_{2}=\theta_{3}=\theta_{4}=0.25$.

It is worth noting that MSE represents the Mean Square Error between the predicted value and the ground truth usually used to deal with regression problems [47]. The dataset randomly generated by Monte-Carlo simulation is not ground truth in itself. But as we explained above, the sleeping rate is a fraction with a value between 0 and 1. Further, the MSE here is a mean square of a small error, it would have no effect on the training but represents our expectation for no outliers. Lastly, the energy consumption, the probabilities of false alarm and detection are all calculated in real-time during the training.

### 4.2. Dataset

As our aim is to minimize the loss function in terms of the SNRs and the parameters for each sensor, labeled data as required in other supervised learning are unnecessary. Our dataset is produced by randomly generating SNRs, sleeping rates, and thresholds for each sensor. Such a dataset suffices to cover a broad range of SNRs that are present in the CRSN and serve to represent the initial points in the training process. Alternatively, we may use the real data obtained from the CRSN measurement. It is expected that this will even yield better results as the neural network will be trained to work on distributions of SNRs closer to reality, and we will consider it in future investigations. Using the Monte-Carlo method to generate 10,000 sets of data which comprise randomly generated SNRs, sleeping rates and thresholds, 80 percent of the data is then split into the training set with the remaining going into the test set. The detailed values for other key parameters are listed in Table 2. According to IEEE 802.15.4/ZigBee standard [48], ZigBee’s transmission distance is 10∼100 m. In this paper, for illustration purposes, the distance *d* between each sensor and FC is set to be the same and equals 70 m, which is within reason. To calculate the transmission energy, $C_{t}$, a free-space path loss model is considered, which leads to the signal attenuation being inversely proportional to the square of *d*, and it is estimated to be 278 nJ [9]. As for the $C_{s}$, based on 1 μs sensing time and the number of samples equal to 5, it is estimated to be 190 nJ [9]. We remark that our method can work for other values of *d*, $C_{s}$, and $C_{t}$.

## 5. Numerical Results and Analysis

We would first validate our proposed neural network in dealing with the multi-dimensional constrained optimization problem. With numerical analysis, the comparison of the proposed approach and the traditional semi-analytic method would be discussed.

### 5.1. Validation of the Designed Neural Network

#### 5.1.1. Loss Iteration

**Figure 3:**
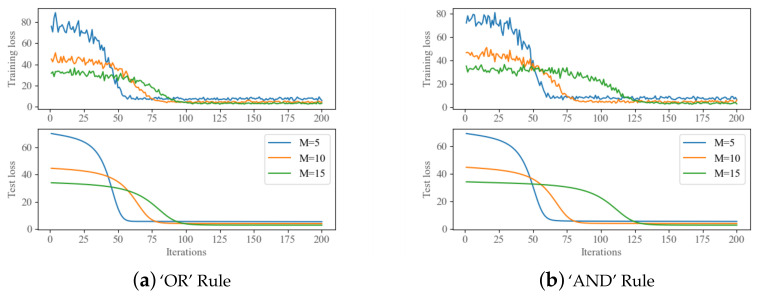
Loss iteration in both training set and test set under ‘OR’ Rule and ‘AND’ Rule.

Figure 3 shows the trend of the training loss as the number of iterations grows under both the ‘OR’ and the ‘AND’ fusion rule. The training loss is always constantly decreasing and constantly fluctuating. Because of our relatively random data set and the loss functionis highly non-linear, some level of volatility is considered reasonable.

With different numbers of sensors, not until the 200 epoch do their loss finally converge. The same trend is shown in the test set with no overfitting, which in turn verifies the rationality of the neural network’s structure design. With the increase of the sensor number, more data in each sample slows down the convergence of the loss function.

#### 5.1.2. Performance Validation

As explained in Section 4, all data for training and testing are randomly generated including SNRs, thresholds, and sleeping rates. That is, each data sample is constructed as $SNR_{1},\ldots ,SNR_{M},\lambda_{1},\lambda_{2},\mu_{1},\ldots ,\mu_{M}$. The purpose of the designed neural network (NN) here is to establish a mapping from sensors’ SNR values to their optimal sleeping rates and two thresholds in energy detection which minimizes the customized loss function comprising the objective function and the constraints. The training process is carried out on the 8000 samples in the training set. For testing, after feeding each sample’s SNRs $\left(SNR_{1},\ldots ,SNR_{M}\right)$ in the test set to our proposed neural network, we can get as outputs a combination of thresholds and sleeping rates $\left(\lambda_{1}^{\prime },\lambda_{2}^{\prime },\mu_{1}^{\prime },\ldots ,\mu_{M}^{\prime }\right)$. We may then compare these results from our proposed NN with the randomly generated sleeping rates and thresholds in terms of the system performances (including *C*, $Q_{d}$, and $Q_{f}$). We can then validate whether the customized loss function had regulated the training direction effectively.

**Figure 4:**
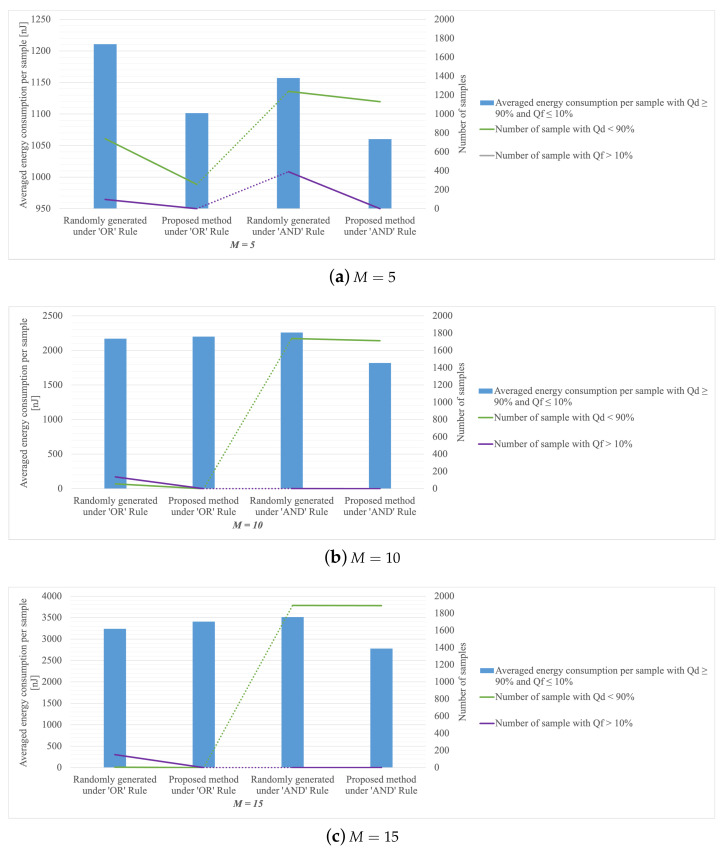
Comparison of generating randomly and selected by NN on the averaged optimal energy consumption per sample for α=0.1 and β=0.9.

With $\pi_{0}=0.5$, the averaged total energy consumption over a CRSN (with 5, 10 and 15 cognitive radios respectively) is depicted in Figure 4 for β=0.9 and α=0.1. The comparison of energy consumption is carried out on samples that satisfy global probabilities requirements under both methods (that is, choose $\lambda_{1},\lambda_{2},\mu_{1},\ldots ,\mu_{M}$ randomly or using the proposed method). We can see that the proposed neural network indeed saves energy consumption with different *M* under either rule. Also in Figure 4, it is shown that the performance in terms of meeting the global probability constraints is generally poor based on the randomly generated sleeping rates and thresholds in the previously noted test set. The proposed neural network with the associated training based on minimizing the customized loss function is shown to be effective, as it achieves remarkable performance in increasing the number of samples that can attain a $Q_{d}$ over 0.9, especially whilst using the ‘OR’ Rule. Furthermore, our proposed training strategy produces impressive results in lowing $Q_{f}$ on samples, suggesting that most bad samples are trained to become good ones, particularly when applying the ‘AND’ Rule.

### 5.2. Proposed Neural Network vs. Traditional Semi-Analytic Method

#### 5.2.1. Complexity Analysis

Assuming each sensor shares the same signal-to-noise ratio and sleeping rate, the traditional semi-analytic method seeks to find only one optimal sleeping rate for all sensors as well as one lower threshold and one upper threshold assuming energy detection through numerical searches [11]. When considering different signal-to-noise ratios among sensors, the size of the numerical search increases exponentially with the number of required parameters. Suppose the number of values available for sleeping rate, lower threshold, and upper threshold are *r*, *s*, *t* respectively, then the search complexity is $o\left({\left(r^{s}\right)}^{t}\right)$. For the data set sources presented in Table 2, without considering the complexity of the comparison after the search, the complexity to search the optimal parameters combination is from $o\left({\left(9^{30}\right)}^{30}\right)$ to $o\left({\left(9^{30}\right)}^{60}\right)$.

In our proposed neural network, after $N_{e}$ epochs and $N_{s}$ samples, the complexity of employing a back-propagation algorithm to train a neural network with *m* inputs, *l* hidden layers, each with $r_{1}$, …, $r_{l}$ neurons, and *n* outputs may be estimated as $o\left(N_{e}N_{s}\left(mr_{1}+\sum_{l-1}^{i=1}r_{i}r_{i+1}+r_{l}k\right)\right)$, but the complexity in the forward route is merely $o\left(mr_{1}+\sum_{l-1}^{i=1}r_{i}r_{i+1}+r_{l}k\right)$, which is much smaller than that of the traditional method.

In the simulation studies where our method is compared against the traditional method, we therefore make the following simplification for the latter method. In order to reduce the search space, we run the numerical search using a single SNR value (Minimum, Averaged, Maximum SNR over all sensors) for the traditional method (hereafter called min-SNR, avg-SNR, max-SNR), and enumerate all values of sleeping rates with a range from 0 to 1 (exclude 0 and 1) in a step size of 0.1. Also enumerated are the upper threshold and lower threshold both with a step size of 1. Only the combination of optimal sleeping rate, upper and lower threshold, which applies to all sensors, that meets the global probabilities requirements and its corresponding system performances are kept, when it comes to calculating the total energy consumption.

#### 5.2.2. Cooperative Spectrum Sensing Performance

When feeding SNRs of multiple sensors to the proposed NN, each sensor would independently enjoy an optimal sleeping rate. For a fair comparison of system performances resulting from different methods, we experimented on a total of 2000 sets of samples in the test set with β=0.9 and α=0.1. Feeding the SNR values of the *M* sensors to the neural network to obtain two thresholds and *M* sleeping rates, we also obtain the *C*, $Q_{d}$, and $Q_{f}$.

**Figure 5:**
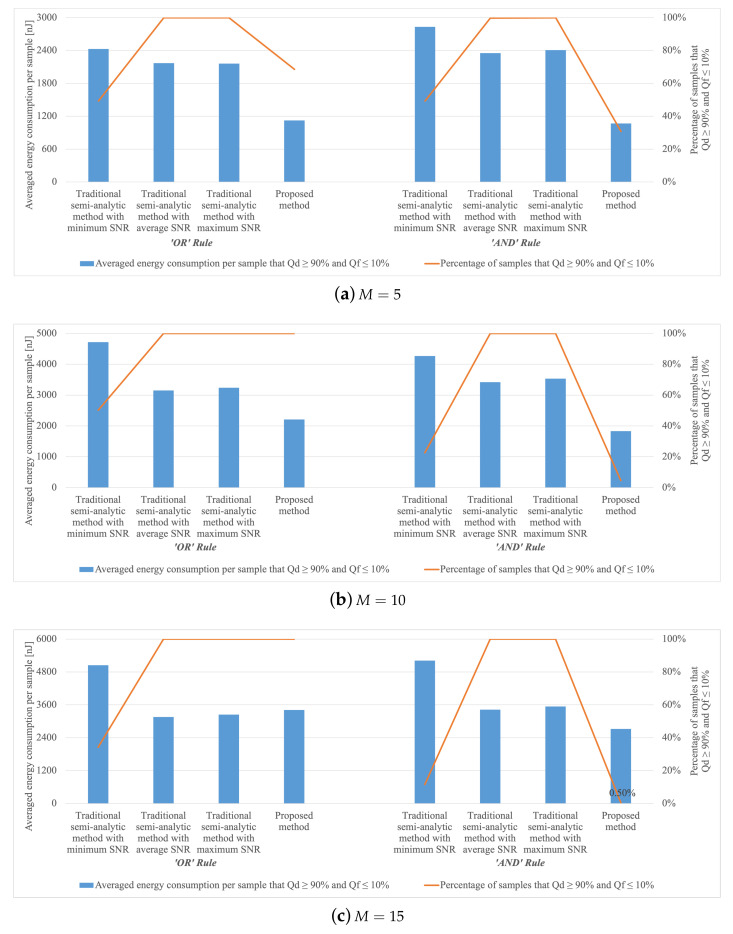
Percentage of samples and average energy consumption under traditional methods (min-SNR, avg-SNR, and max-SNR) and proposed method meeting α=0.1 and β=0.9.

Given the probability of PU absence is 0.5, we count the number of samples in the test set that can meet system performance requirements ($Q_{d}\geq 90\%$ and $Q_{f}\leq 10\%$) under each method with a different number of sensors, as shown in Figure 5.

Under the ‘OR’ fusion rule, it seems that the traditional methods with avg-SNR and max-SNR may outperform our proposed method in terms of the percentage of samples without violating the global probabilities constraints when M=5. Yet, with all different *M*, our proposed method yields a lower or almost the same average energy-consumption-per-sample for more samples satisfying the constraints, in comparison with the traditional methods.

Under the ‘AND’ fusion rule, the avg-SNR seems to be the best, followed by the max-SNR, while the min-SNR always consumes most but few satisfy the global probability constraints. Our proposed neural network doesn’t perform well on the proportion of samples that do not violate the global probabilities constraints, but always consumes the least energy, which is even lower than half of that under the traditional method when M=5,10.

Obviously, the traditional method extremely relies on the density of the exhaustive search, the smaller the step size, the easier it is to find good values. Due to the differences in SNRs, using one single sleep rate for all sensors is far from optimal in terms of energy efficiency. This is clear as the traditional method demonstrates under both rules that its energy consumption is often higher than that of our proposed approach.

In sum, the proposed method can yield lower or comparable energy consumption for samples satisfying the constraints compared with traditional numerical-search methods. One way to improve the number of samples satisfying the constraints is to augment the proposed method by running the traditional method alongside and the additional samples satisfying the constraints obtained from the traditional method can be included. If both methods yield samples that satisfy the constraints, we may select the ones with lower energy consumption. Therefore, these methods may complement each other and bring about further improvement.

#### 5.2.3. Robustness Comparision

**Figure 6:**
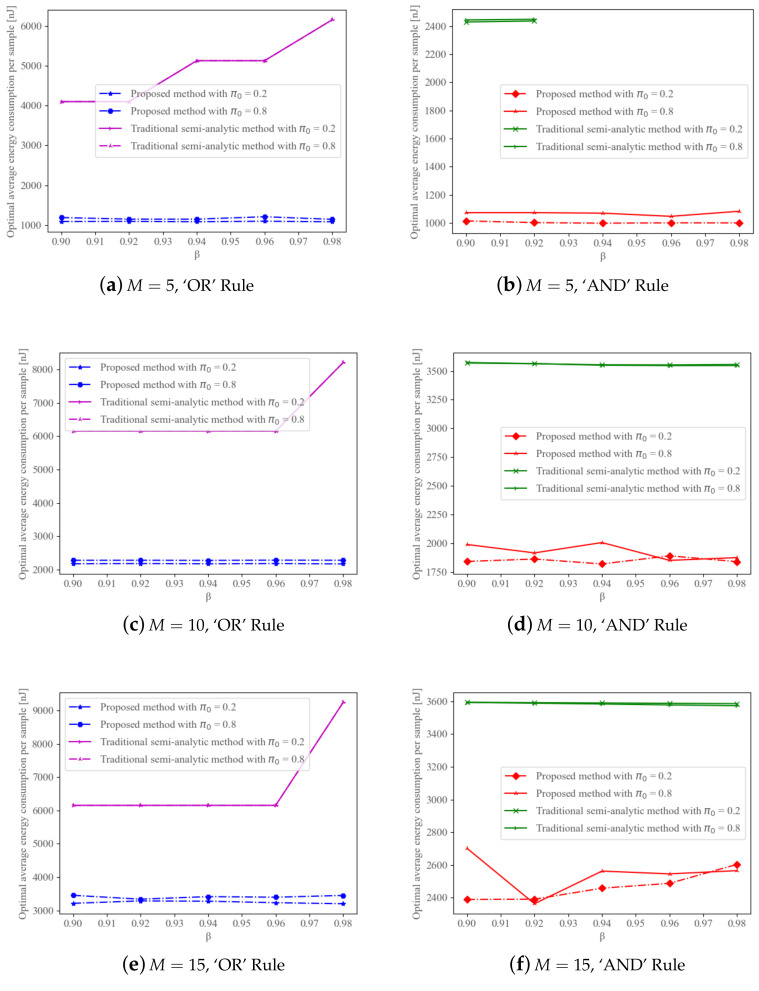
Energy consumption per sample versus the probability of detection under two methods (avg-SNR and proposed method) with α=0.1.

The advantage of our proposed neural network in terms of robustness is shown in Figure 6. The number of cognitive radios is also the same as before, that is, $\left\{\begin{array}{c} 5,10,15 \end{array}\right\}$. The boundary of the probability of false alarm is 10% and the probability of detection is set to $\left\{\begin{array}{c} 90\%,92\%,94\%,96\%,98\% \end{array}\right\}$. We let $\pi_{0}$ adopt two values including $\left\{\begin{array}{c} 0.2,0.8 \end{array}\right\}$ to correspond to low and high probabilities of PU absence, respectively. But to save search time, we further simplify the traditional semi-analytical approach to only enumerating the sleeping rate, letting it use the same lower and upper thresholds from the training results of our proposed neural network.

The optimal average energy consumption per sample versus the probability of detection is depicted in Figure 6. We can see that while using the traditional method, the energy consumption usually increases greatly with the probability of detection under the ‘OR’ Rule. In fact, even if the energy consumption doesn’t increase under the ‘AND’ Rule, with the growth of β, only fewer and fewer fractions of the samples can satisfy the constraints on global probabilities. Especially when M=5, the traditional method can find no parameters’ combination anymore, once β>92%. The neural network consumes the least energy even as the global requirement changes and can always find the parameter combination without being affected. We can further infer that our proposed neural network is robust to changes in β while the traditional method’s performance is critically dependent on β.

Note that this paper only considers a distributed spectrum sensing with one FC. The energy-efficient decentralized spectrum sensing is not considered. For energy efficiency, a sleeping and censoring method is introduced and we simply focus on the ‘AND’ and ‘OR’ rules in this case. Extension to other fusion rules and other energy-efficient CSS schemes is a subject of future work.

## 6. Conclusions

In this paper, the cooperative spectrum sensing (CSS) utilizing a sleeping and censoring scheme is investigated and optimized in terms of energy efficiency using neural network. The CSS parameters, including the lower threshold, upper threshold, and sleeping rates for the sensors, are determined through training on the neural network with the goal of reducing the total energy consumption. Specifically, for a CSS system with multiple sensors, the proposed neural network can yield good sleeping rates and thresholds with reduced energy consumption, as well as promise a certain level of global false alarm and detection probabilities. The novelty of the proposed approach is that such an energy efficiency optimization problem, which involves multiple parameters and constraints, can be handled as a multi-output regression problem with a customized loss function that can be solved by the neural network and the proper learning. Different from the traditional semi-analytic method, our method based on neural network provides feasible means to determine the optimal system parameters to minimize energy consumption in a practical radio environment in which different sensors experience different SNRs and channel gains. The simulations show that the proposed method can yield lower energy consumption than traditional methods in selected scenarios. Furthermore, the proposed method can be complemented with other existing methods to yield better CSS performance in terms of energy efficiency and detection rate.

## Figures and Tables

**Table 1:** Metrics for global decisions under ‘OR’ rule and ‘AND’ Rule.

Rule	Global Probability of Detection	Global Probability of False Alarm
‘OR’ Rule	$Q_{d}^{OR}=1-\Pi_{j=1}^{M}\left[1-\left(1-\mu_{j}\right)P_{d,j}\right]$	$Q_{f}^{OR}=1-\Pi_{j=1}^{M}\left[1-\left(1-\mu_{j}\right)P_{f}\right]$
‘AND’ Rule	$Q_{d}^{AND}=1-\Pi_{j=1}^{M}\left[1-\left(1-\mu_{j}\right)\left(1-\sigma_{1,j}-P_{d,j}\right)\right]$	$Q_{f}^{AND}=1-\Pi_{j=1}^{M}\left[1-\left(1-\mu_{j}\right)\left(1-\sigma_{0,j}-P_{f}\right)\right]$

**Table 2:** Detailed parameters for generating a data set.

Symbol	Description	Value
*N*	Number of samples	5
*M*	Number of sensors/cognitive radios	5, 10, 15
Average SNR	Average signal-to-noise ratio of the sensors	$\left[0,15\right]$ dB
$\mu_{j}$	Sleeping rate of the j-th sensor	$\left(0,1\right)$
$\lambda_{1}$	Lower threshold in Energy Detection	$\left[0,30\right]$
$\lambda_{2}$	Upper threshold in Energy Detection	$\left[\lambda_{1},60\right]$
*d*	Distance between sensors and FC	70 m
$C_{s}$	Sensing energy in *N* Samples	190 nJ
$C_{t}$	Transmission energy in d m distance per bit decision	278 nJ

## Data Availability

Data is contained within the article.

## Abbreviations

The following abbreviations are used in this manuscript:

FCC	Federal Communications Commission
DSA	Dynamic Spectrum Access
CR	Cognitive Radio
PU	Primary User
SU	Secondary User
ED	Energy Detection
CRSN	Cognitive Radio Sensor Network
CSS	Cooperative Spectrum Sensing
SH	Spectrum Hole
ML	Machine Learning
DL	Deep Learning
NN	Neural Network
RL	Reinforcement learning
